# Survival outcomes in patients with oropharyngeal cancer treated with carboplatin/paclitaxel and concurrent radiotherapy

**DOI:** 10.1186/s40463-016-0163-1

**Published:** 2016-10-10

**Authors:** M. Roskies, E. Kay-Rivest, M. A. Mascarella, K. Sultanem, A. Mlynarek, M. Hier

**Affiliations:** 1 Department of Otolaryngology-Head and Neck Surgery, Jewish General Hospital, McGill University, Montreal, Quebec Canada; 2 Department of Radiation Oncology, Jewish General Hospital, McGill University, Montreal, Quebec Canada

**Keywords:** Oropharyngeal squamous cell carcinoma, Carboplatin, Radiotherapy, Survival

## Abstract

**Background:**

A commonly employed treatment for advanced staged oropharyngeal squamous cell carcinoma (OPSCC) is concurrent radiation and chemotherapy with cisplatin as the gold standard. Carboplatin is reported to have the same radiopotentiation properties and a superior side effect profile; however, its use in head and neck cancer has been limited due to the paucity of data and reported hematologic side effects. In this study, we describe our institution’s experience with carboplatin, paclitaxel and radiation in the treatment of oropharyngeal squamous cell carcinoma over a 10 year period.

**Methods:**

A retrospective chart review of patients aged 18 to 80 years old with stage III-IV OPSCC treated with weekly carboplatin, paclitaxel and intensity modulated radiotherapy (IMRT) was performed. Data collected included patient demographics, tumor location and stage and survival outcomes. In addition, we noted treatment morbidities according to the Radiation Therapy Oncology Group (RTOG) scoring criteria scale. The data was analyzed using the student’s *t*-test and analysis of variables.

**Results:**

Over a 10 year period, 160 patients received chemoradiation with carboplatin and paclitaxel for OPSCC. One-hundred-four and 65 patients were followed for at least 3 and 5 years, respectively. Overall survival for all patients was 81.7 and 70.7 % at 3 and 5 years, respectively. Disease free survival and locoregional recurrence-free survival at 5 years was 64.6 and 89.2 %, respectively. There was no association of survival with stage, regional nodal status or subsite. The most common side effect attributed to treatment was acute dysphagia (75.25 %) followed by odynophagia, xerostomia and hypogeusia. Hospitalizations and non-hospitalization emergency department visits attributed to treatment totalled 33 and 11, respectively. Hematologic toxicities greater than grade II were: 11.9 % neutropenia, 6.3 % anemia, 1.8 % thrombocytopenia. The incidence of febrile neutropenia was 5.0 % (8/160).

**Conclusion:**

In conclusion, the overall, disease-free and locoregional recurrence-free survival of patients treated with carboplatin and radiotherapy for advanced stage OPSCC parallels what has been described in the literature for cisplatin, with an acceptable side effect profile.

## Background

The rising incidence of oropharyngeal squamous cell carcinoma (OPSCC) has centered a focus in the literature on appropriate therapies. One of the preferred recommendations of the National Comprehensive Cancer Network (NCCN) for advanced stage OPSCC is concurrent high dose cisplatin and radiotherapy. This aggressive therapeutic strategy is a response to many patients presenting with locally advanced stage and, as a result, adverse events from cisplatin are common, including nephrotoxicity and ototoxicity. Additionally, the accompanying drug-resistant nausea and vomiting often-times limits the duration and dose of therapy. Not surprisingly, the overall 5-year survival rate for locally advanced OPSCC mirrors other subsites of the head and neck and ranges between 39 and 61 % [1].

At our institution, the preferred chemotherapeutic strategy employed for patients with OPSCC not enrolled in randomized clinical trials is carboplatin combined with paclitaxel. The decision to use carboplatin instead of cisplatin for eligible patients was made after a landmark phase II trial demonstrated that carbotaxol protocols were well tolerated without compromising survival as compared to historical controls [2]. Carboplatin is a structurally similar agent to cisplatin and appears to have a good clinical response with less morbidity. Despite the widespread use of cisplatin, there is a paucity of research focused on the outcomes in patients with stage III and IV OPSCC undergoing treatment with carboplatin. The purpose of our study was to evaluate survival and adverse events in patients with advanced stage OPSCC undergoing concurrent CRT using carboplatin and paclitaxel.

## Methods

### Design

After obtaining Research Ethics Board approval, a retrospective case series review was performed. Patients undergoing concurrent carboplatin, paclitaxel and radiotherapy for advanced (stage III or IV) oropharyngeal squamous cell carcinoma (OPSCC) at a tertiary care academic centre from 2001 to 2012 were included. All received carboplatin (dose AUC) and paclitaxel (35–40 mg/m2) over 6 weeks concomitant with external beam radiotherapy using Volumetric Modulated Arc Therapy with integral concomitant boost (Gross Total Volume is prescribed 67.5 Gy in 30 fractions over 6 weeks and clinical target volume is prescribed 54–60 Gy in 30 fractions). Only patients undergoing treatment for curative intent were included. Patients enrolled in clinical trials involving surgery or alternative chemotherapeutic regimens, including cisplatin, or with recurrent disease were excluded. With patient consent, submandibular gland transfer to the submental space was performed prior to CRT to reduce radiation-induced xerostomia [3]. The gland was transferred to an area that is shielded during radiation therapy. Patients with bilateral or contralateral nodal, any nodes in level I or primary cancers of the base of tongue crossing the midline were excluded from the procedure.

### Outcomes

Medical records were reviewed for demographic profile, tumor location and staging, p16 status and adverse events. In addition, 3 and 5 year survival outcomes, including overall survival (OS), loco-regional free survival (LRFS) and disease free survival (DFS) were analyzed. LRFS and DFS were based on radiologic and pathologic findings as guided by the follow-up recommendations of the NCCN for head and neck cancer. This includes follow-up visits with a head and neck surgeon and radiation oncologist at the first 6 to 8 weeks following completion of treatment. Computed tomography (CT) imaging of the head and neck was performed at 3 months following the completion of CRT to assess treatment response. Suspicious symptoms, lesions on physical exam or on imaging were further evaluated with tissue biopsies as judged clinically. Furthermore, CRT associated morbidities were noted in accordance to the Radiation Therapy Oncology Group (RTOG) acute radiation morbidity scoring criteria scale. Emergency room visits or hospitalizations associated with chemo-radiotherapy were also recorded.

### Analysis

Survival outcomes were analyzed using Kaplan-Meier survival curves. The 3 and 5 year survival outcomes based on age, gender and TNM stage. Chi-squared testing was used to compare categorical data and the independent *t*-test for continuous data. A *p*-value of less than 0.05 was considered statistically significant. All data was computed using Medcalc (version 12.2).

## Results

A total of 160 patients with an advanced stage OPSCC were analysed during the 10 year study period. One-hundred-five and 65 patients were followed for at least 3 and 5 years, respectively. The average age of the population was 61.1 years (±9.6 years), with a male predominance (72.5 %). The majority of tumors originated from the palatine tonsils (84/160) or base of tongue (70/160). Thirty-eight patients (24 %) had stage III disease and 142 (76 %) non-metastatic stage IV disease. Table 1 lists the patient characteristics.

**Table 1:** Patient and Tumor Characteristics

Number of patients	160
Age	6.1 (±9.6)
Gender (% male)	72.5 % (116/160)
Disease site	
Tonsil	84
DOT	70
Soft palate	3
Oropharynx (other)	3
Tumor	
T1	54
T2	42
T3	35
T4	29
Nodal Status	
N0	12
NI	29
N2 (N2a, N2b, N2c)	108 (67, 14, 27)
N3	11
TNM Stage	
Stage III	38
StagelV	122
Salivary gland transfer	38

**Fig. 1:**
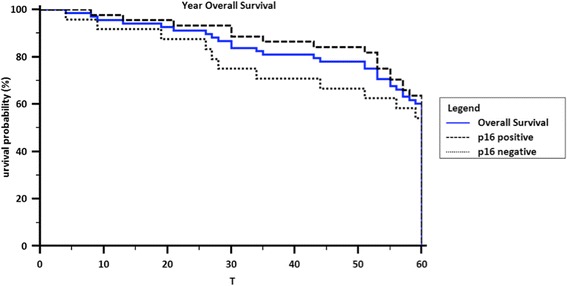
Five Year Overall Survival in patients with advanced stage oropharyngeal carcinoma

**Fig. 2:**
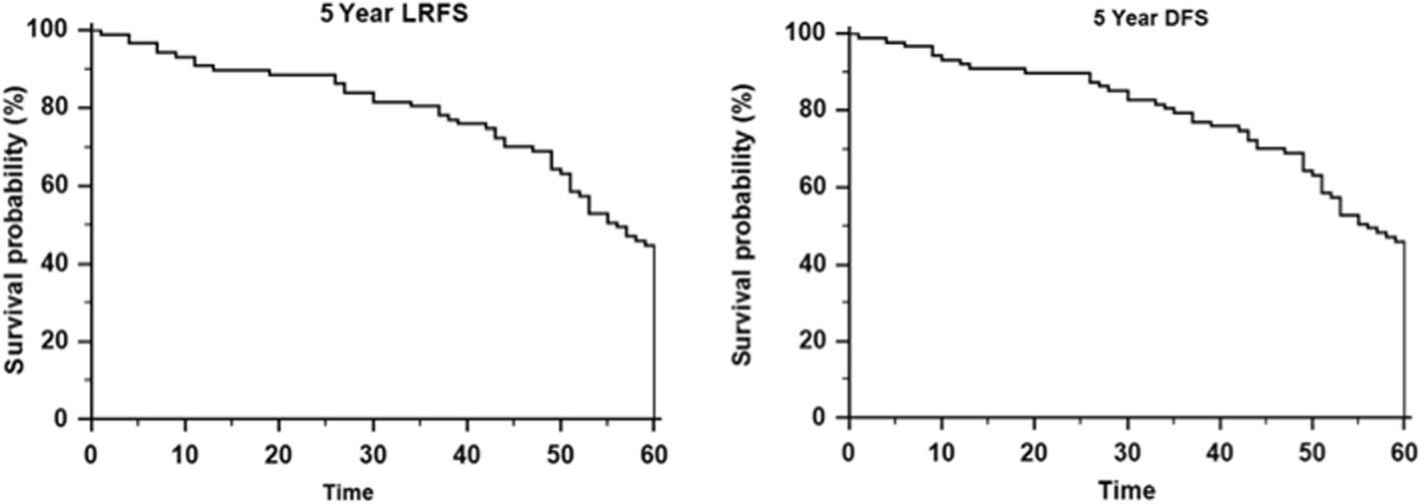
Loco-regional and disease free survival outcomes

The overall survival for all patients was 81.7 and 70.7 % at 3 and 5 years, respectively (Fig. 1). DFS and LRFS survival at 5 years was 64.6 and 89.2 %, respectively (Fig. 2). There was no association of survival with stage, regional nodal status or subsite (*p* > 0.05).

**Fig. 3:**
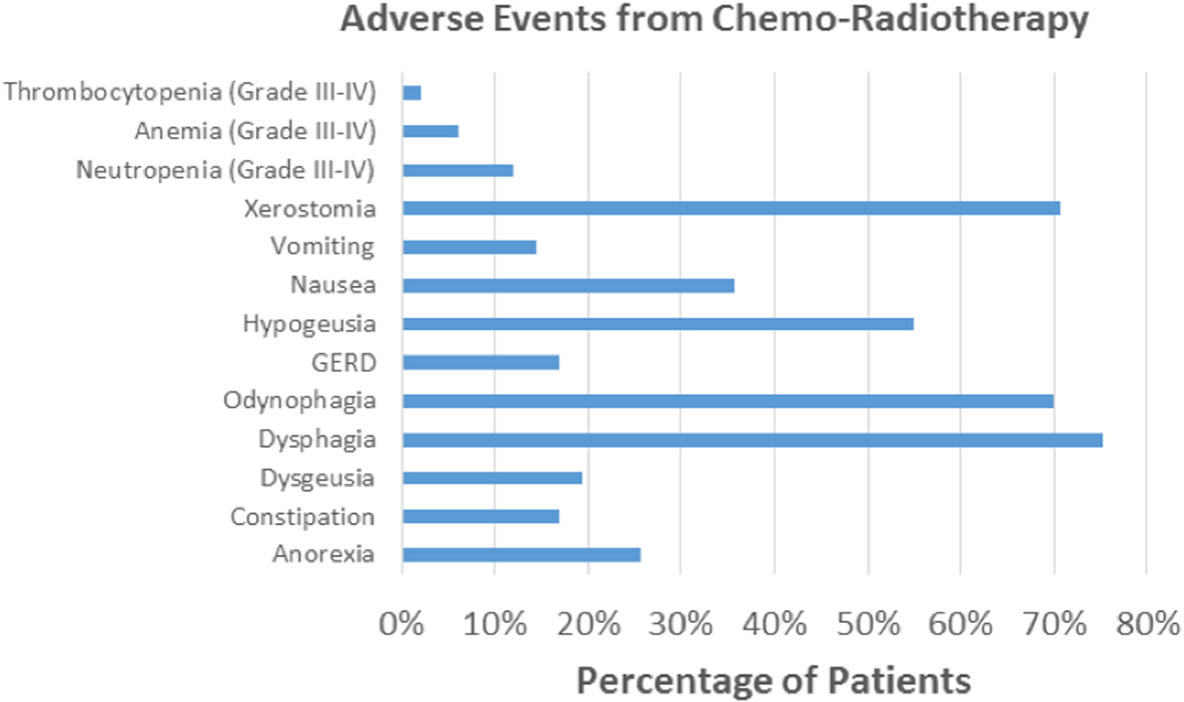
Adverse events in patients treated with carboplatin, paclitaxel and radiotherapy

**Fig. 4:**
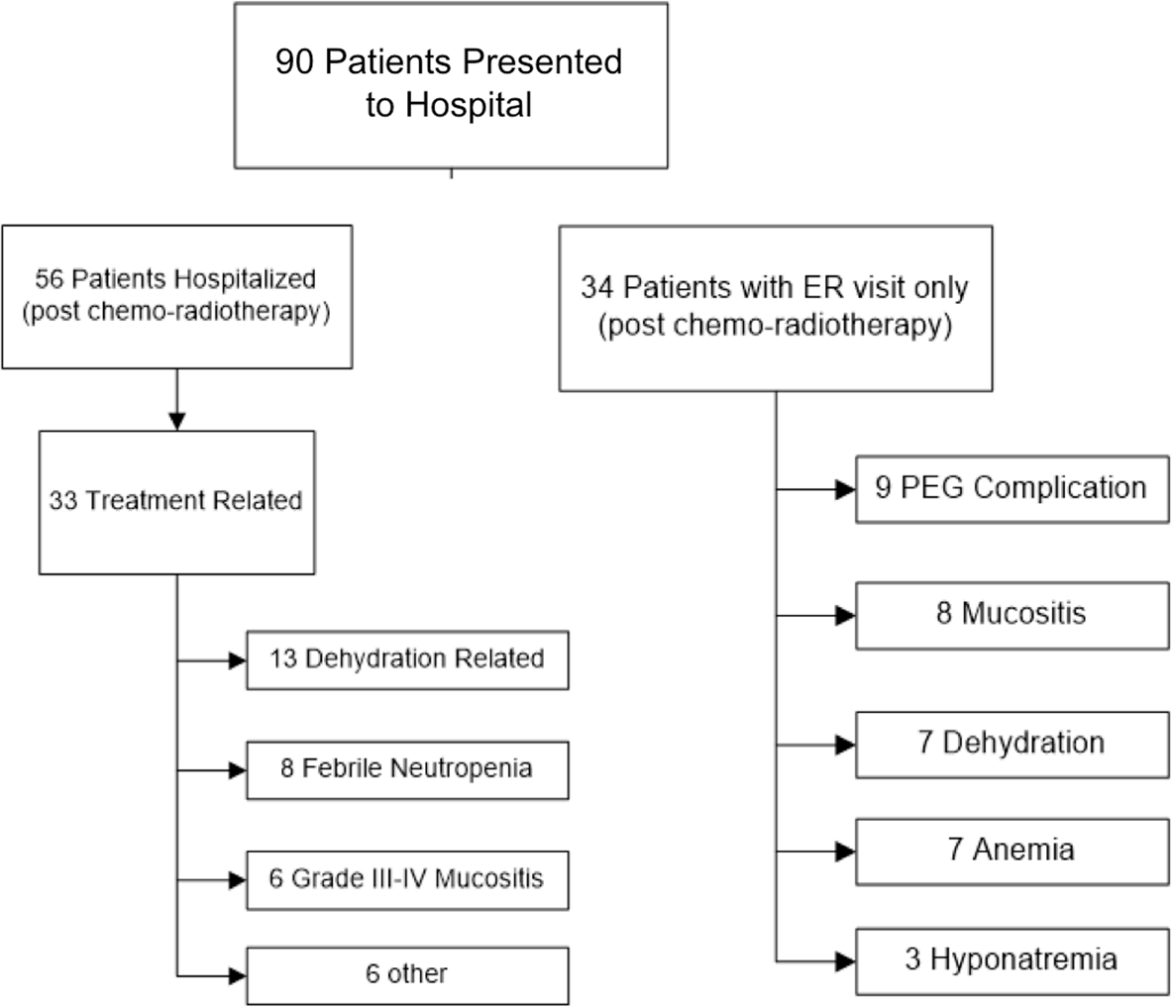
Emergency department visits and hospitalizations

The most common adverse event associated with treatment was acute dysphagia (75.25 %) followed by odynophagia, xerostomia and hypogeusia (Fig. 3). Ninety patients returned to the emergency room with CRT-related complaints, of which 34 required hospitalization. The main reasons for hospitalization included severe dehydration, febrile neutropenia, grade III/IV mucositis and PEG tube complications. Major adverse events (grade III or IV) consisted of: 11.9 % neutropenia, 6.3 % anemia, 1.8 % thrombocytopenia and 3.8 % mucositis (Fig. 4).

## Discussion

Platinum analogues are among the primary agents used to treat numerous solid tumors, including ovarian, lung, head and neck, as well as bladder cancer, among others. Cisplatin and carboplatin are the two most commonly used platinum-based agents. They act in similar fashions, as non-classical alkylating agents that bind to cellular DNA and form crosslinks [4]. Cisplatin was the first platinum analogue introduced for oncologic therapy. It carries a significant toxicity profile, which includes nausea and vomiting, and risk of renal dysfunction, neurotoxicity and ototoxicity [4]. Soon after its development, carboplatin, was developed which was believed to have a milder side effect profile. It is thought to cause less gastrointestinal, renal and neural toxicity. However, it does carry a risk of bone marrow suppression, thrombocytopenia in particular.

In this study, the OS, DFS and LRFS of patients with advanced stage OPSCC treated with concurrent carboplatin/paclitaxel and radiation parallels outcomes described in the literature for cisplatin use. Phase II trials have demonstrated 2-year overall survival of 66.6 % for stage III/IV resectable oro/hypopharyngeal treated with cisplatin, paclitaxel and radiation therapy [5] and 37.8 % for those deemed unresectable [6]. Furthermore, the side effect profile of carboplatin appears superior. There are few articles in the literature describing experiences with carboplatin/paclitaxel and radiotherapy treatment for advanced stage OPSCC, as most institutions employ cisplatin as their primary chemotherapeutic agent [7–9].

Ezra et al. first demonstrated the superiority of concomitant carboplatin to radiation in advanced stage oropharyngeal carcinoma [10]. Three-year overall survival for patients receiving carboplatin and 5-fluorouracil in this phase III study was 51 %, less than the 81.7 % recorded in our series. Additionally, the rates of mucositis (71 %) and thrombocytopenia (5.5 %) were higher than our series, whereas anemia (2.8 %) and neutropenia (3.7 %) were lower.

Two trials have compared cisplatin and carboplatin in a prospective randomized fashion. The first, performed by Forastiere et al. compared cisplatin plus fluorouracil and carboplatin plus fluorouracil versus methotrexate in all advanced squamous-cell carcinomas of the head and neck [9]. Their trial showed greater ototoxicity and renal toxicity with cisplatin compared to carboplatin. Interestingly, they also found higher hematologic toxicity in the cisplatin group. Although the clinical response rate was higher for cisplatin compared to carboplatin, they found similar response duration and median survival with both agents. However, of greater influence was the trial by De Andrés et al., which was interrupted early after the enrolment of only 95 patients, due to evidence of superiority of cisplatin in terms of response rate, disease free survival and overall survival [9, 11]. This group’s patient population differs from this current retrospective study, as they only included stage IV non-metastatic patients.

Another phase II trial, which studied the use of single-agent carboplatin with concurrent radiotherapy showed 66 % complete remission and 98 % overall response rate. Fifty-three of 56 patients remained disease-free with a median survival over 25 months [12]. Despite the low enrolment, it appears that carboplatin, in combination with radiation therapy, can achieve similar outcomes to cisplatin with a more favorable side effect profile. Agarwala et al. performed a phase II trial in patients considered inoperable with advanced OPSCC treated patients with concurrent carboplatin, paclitaxel and radiation therapy. In these patients, the 5-year progression free survival was 36 % and the 5-year overall survival was 35 %. They concluded that this regimen was safe for OPSCC patients with advanced disease. Finally, another phase II study was performed by Chougule et al. also studied concurrent chemoradiotherapy with weekly paclitaxel and carboplatin in advanced head and neck cancers, and found good curative potential and lower rates of toxicity than with cisplatin.

The recognition of HPV-associated OPSCC represents an important advance in the field of head and neck cancers. It confers an important survival advantage compared to HPV-negative cancer. Unfortunately, this dataset was lacking a significant amount of p16 staining results as routine reporting of HPV-related OPSCC began in 2009 at our centre. For the purpose of this study, we have omitted a discussion of HPV as it relates to our outcomes, but are aware of the inherent confounding this introduces.

## Conclusion

Survival outcomes for patients with stage III and IV non-metastatic OPSCC treated with concurrent carboplatin/paclitaxel and radiotherapy parallels protocols involving cisplatin, with a better side effect profile. A prospective study is needed to clarify the role of concurrent CRT with carboplatin and paclitaxel in this population.
